# Expression of ABCB1, ABCB5, and ABCG2 Transporters in Human Renal Cell Carcinoma and Their Underlying Signaling Pathways

**DOI:** 10.3390/cimb48070739

**Published:** 2026-07-21

**Authors:** Anna Vass, József Király, Erzsébet Szabó, Gábor Kónya, Ali Shammas, Krisztián Szegedi, Balázs Dezső, Éva Juhász, Gábor Halmos, Zsuzsanna Szabó

**Affiliations:** 1 Department of Biopharmacy, Faculty of Pharmacy, University of Debrecen, 4002 Debrecen, Hungarykiraly.jozsef@pharm.unideb.hu (J.K.); konya.gabor@pharm.unideb.hu (G.K.); 2 Doctoral School of Pharmaceutical Sciences, University of Debrecen, Nagyerdei krt. 98, 4032 Debrecen, Hungary; shammas.ali@pharm.unideb.hu; 3 Department of Pharmacodynamics, Faculty of Pharmacy, University of Debrecen, 4002 Debrecen, Hungary; erzsebet.szabo@med.unideb.hu; 4 HUN-REN-DE Pharmamodul Research Group, University of Debrecen, 4002 Debrecen, Hungary; 5 Department of Urology, Faculty of Medicine, University of Debrecen, 4032 Debrecen, Hungary; szegedi.krisztian@med.unideb.hu; 6 Department of Pathology, Faculty of Medicine, University of Debrecen, 4032 Debrecen, Hungary; bdezso@med.unideb.hu; 7 Department of Pediatrics, Faculty of Medicine, University of Debrecen, 4032 Debrecen, Hungary; juhasze@med.unideb.hu

**Keywords:** RCC, ccRCC, sunitinib, CAKI-2, A-498, ABC transporters, ABCB1, ABCB5, ABCG2

## Abstract

Renal cell carcinoma (RCC) is frequently resistant to tyrosine kinase inhibitors (TKIs) such as sunitinib, limiting therapeutic efficacy. ATP-binding cassette (ABC) transporters, including ABCB1, ABCB5, and ABCG2, are important transporters implicated in multidrug resistance, influencing drug efflux and tumor progression. We aimed to evaluate the expression of ABCB1, ABCB5, and ABCG2 in human RCC tissues and human renal cancer cell lines CAKI-2 and A-498, and to investigate their potential association with sunitinib resistance and associated signaling pathways. Twenty paired tumorous and adjacent non-tumorous human kidney tissue samples were analyzed for ABC transporter gene expression using qRT-PCR. RCC cell lines CAKI-2 and A-498, including sunitinib-resistant derivatives, were treated with 40 µM sunitinib. The levels of transporters and key signaling proteins were assessed by Western blot. ABCG2 was consistently higher in tumorous tissues, and ABCB1 and ABCB5 showed grade-dependent increases in tumors. Resistant cells exhibited elevated ABCB1 and dynamic ABCB5 and ABCG2 expression patterns compared to sensitive cells, indicating an association between altered transporter expression and the resistant phenotype. Sunitinib treatment modulated signaling pathways, with differential activation of PI3K/Akt, NF-κB, and MAPK/ERK observed in sensitive versus resistant cells. Our findings suggest that altered ABCB1 and ABCG2 expression may be associated with the development of sunitinib resistance in RCC and may be linked to changes in key survival signaling pathways. These findings provide a basis for future functional studies investigating the role of ABC transporters in sunitinib resistance and may contribute to the development of personalized therapeutic strategies in RCC.

## 1. Introduction

Worldwide, renal cell carcinoma (RCC) is the sixth most commonly diagnosed cancer in men and the tenth most commonly diagnosed cancer in women, accounting for ap-proximately 5% and 3% of all cancer diagnoses, respectively [1,2,3]. Despite improvements in diagnostic imaging, RCC is often detected incidentally, and its global mortality remains high, with approximately 140,000 deaths annually [2,3].

Most patients are diagnosed at a localized stage, where surgical resection (partial or radical nephrectomy) is the standard treatment [3,4]. However, recurrence or progression to metastatic RCC (mRCC) can occur in a significant proportion of patients within five years of initial treatment [4]. mRCC is largely resistant to conventional chemotherapy and hormonal therapies, while traditional immunotherapies offer only limited benefit. As the molecular mechanisms of RCC have become better understood, targeted therapies have gained prominence [5]. Among them, tyrosine kinase inhibitors (TKIs) such as sunitinib have shown clinical benefit, although treatment resistance and disease relapse remain major challenges [6]. In addition, sunitinib is associated with frequent adverse effects and often leads to relapse due to drug resistance [7,8]. A key contributor to treatment failure is multidrug resistance (MDR), which may be intrinsic or acquired. One of the mechanisms associated with MDR is the altered expression of ATP-binding cassette (ABC) transporters, which may reduce intracellular drug accumulation by actively exporting chemotherapeutic agents [9,10,11].

ABC transporters are a large, evolutionarily conserved protein family involved in transporting endogenous and exogenous compounds. While they play a protective role in healthy tissues—particularly in the kidney and gastrointestinal tract—this same function may contribute to chemotherapy failure by reducing intracellular drug concentrations in tumor cells. They are frequently overexpressed, even in drug-sensitive cancers, suggesting a potential role in tumor progression and treatment resistance [11,12,13,14]. The role of ABCB1 (ATP binding cassette subfamily B member 1) in drug resistance has been well documented [15,16,17]. Overexpression of ABCB1, a transporter with broad substrate specificity, has been demonstrated in kidney tumors, likely due to the kidney’s excretory function and constant exposure to xenobiotics [18,19,20]. ABCB5 (ATP binding cassette subfamily B member 5), a lesser-known P-glycoprotein family member, has also been implicated in MDR. It has been detected in melanoma, oral squamous cell carcinoma, and tissues of neuroectodermal origin [14,21]. Its expression in kidney cortex and medulla suggests a role in renal detoxification, particularly in xenobiotic elimination [14]. ABCG2 (ATP binding cassette subfamily G member 2), another important ABC transporter, regulates reactive oxygen species (ROS) homeostasis and contributes to multidrug resistance by exporting various anticancer agents [22]. These transporters were selected for the present study because their altered expression has been associated with resistance to TKI agents, including sunitinib, primarily through their role in drug efflux and the regulation of intracellular drug accumulation [21,22].

These transporters often share overlapping substrate specificity and can be co-expressed, suggesting potential functional compensation among them. Inhibition of one ABC transporter can result in compensatory upregulation or increased activity of another, it is plausible that they influence each other’s function [23]. Moreover, intracellular signaling pathways such as PI3K/Akt (Phosphoinositide 3-kinase/Protein kinase B), MAPK/ERK (Mitogen-activated protein kinase/Extracellular signal-regulated kinase), and Wnt/β-catenin (Wingless-Type MMTV Integration Site/β-catenin) have been shown to regulate ABC transporter expression, linking them to both drug resistance and tumor progression. NF-κB signaling has also been implicated in the regulation of ABC transporter expression and the development of multidrug resistance. Moreover, extensive crosstalk exists between NF-κB and the PI3K/Akt and MAPK/ERK pathways, suggesting that coordinated activation of these signaling networks may contribute to tumor progression and therapeutic resistance in RCC [24,25,26]. In addition to their role in drug resistance, ABC transporters are involved in transporting tumor-promoting molecules and mediating protein–protein interactions that influence tumor aggressiveness and prognosis [11]. Given the dysregulation of ABCB1, ABCB5, and ABCG2 in various cancers, including RCC, it is plausible that they influence each other’s function, studying their co-expression and interplay could shed light on new mechanisms of resistance and disease progression [11,27,28].

Although much of the current evidence is correlational, several studies have linked abnormal ABC transporter expression to more aggressive tumor features, including stage, size, metastatic potential, and prognosis. Whether these proteins are merely markers of aggressiveness or active contributors to tumor progression remain unclear [11]. Despite the kidney’s prominent role in drug elimination, data on ABC transporter expression in both healthy and malignant renal tissue remain limited. Very few studies have investigated ABCB1, ABCB5, and ABCG2 specifically in renal tumors. Therefore, in this study, we aimed to analyze the expression of these transporters at tissue and cellular levels. Through two in vitro human kidney cancer cell lines, CAKI-2 and A-498, we examined the effects of sunitinib on transporter expression and their potential association with resistance-related molecular alterations [11].

In the present study, we investigated the gene and protein expression of ABCB1, ABCB5, and ABCG2 in human RCC tissues and in sensitive and sunitinib-resistant RCC cell lines. Furthermore, we examined changes in transporter expression following sunitinib treatment together with selected signaling pathways involved in cellular survival and stress responses. Our aim was to characterize transporter expression profiles in these experimental models and to improve our understanding of the molecular alterations associated with RCC and sunitinib treatment. With this research, we aim to contribute to the development of personalized and targeted therapeutic approaches for RCC patients.

## 2. Materials and Methods

### 2.1. RCC Tissue Samples

In our study, 20 pairs of tumorous and adjacent healthy human kidney tissue samples were included. The 40 paired samples studied were collected from patients who underwent surgery at the Department of Urology, University of Debrecen. The samples were collected with the approval of the Medical Ethics Committee of the University of Debrecen (UD REC/IEC 4831-2017). The mean age of the patients was 64 years, with a range of 45–83 years.

Histological examination of the samples was performed by an expert pathologist according to the TNM staging system. The surgically obtained tissue samples were stored at −80 °C until further use. The WHO grading system was used to determine histological grade. T staging was applied to evaluate local invasion, and lymph node status was recorded as positive or negative.

### 2.2. Cell Lines

The human clear cell renal cell carcinoma (ccRCC) cell lines A-498 (ATCC HTB-44) and CAKI-2 (ATCC HTB-47) used in this study were obtained from the American Type Culture Collection (ATCC, Rockville, MD, USA). The cells were cultured in T75 flasks in IMDM (Iscove’s Modified Dulbecco’s Medium, Biosera, Cholet, France), supplemented with 10% fetal bovine serum (FBS; Biosera, Cholet, France) and antibiotics (100 U/mL penicillin and 100 µg/mL streptomycin), at 37 °C in a 5% CO_2_ incubator, and passaged at least twice a week.

### 2.3. Chemicals

Sunitinib was purchased from Santa Cruz Biotechnology (Dallas, TX, USA). It was dissolved in dimethyl sulfoxide (DMSO) to prepare a stock solution at a concentration of 251 mM, and stored at −20 °C.

### 2.4. Sunitinib-Resistant Cell Lines

**Figure 1:**
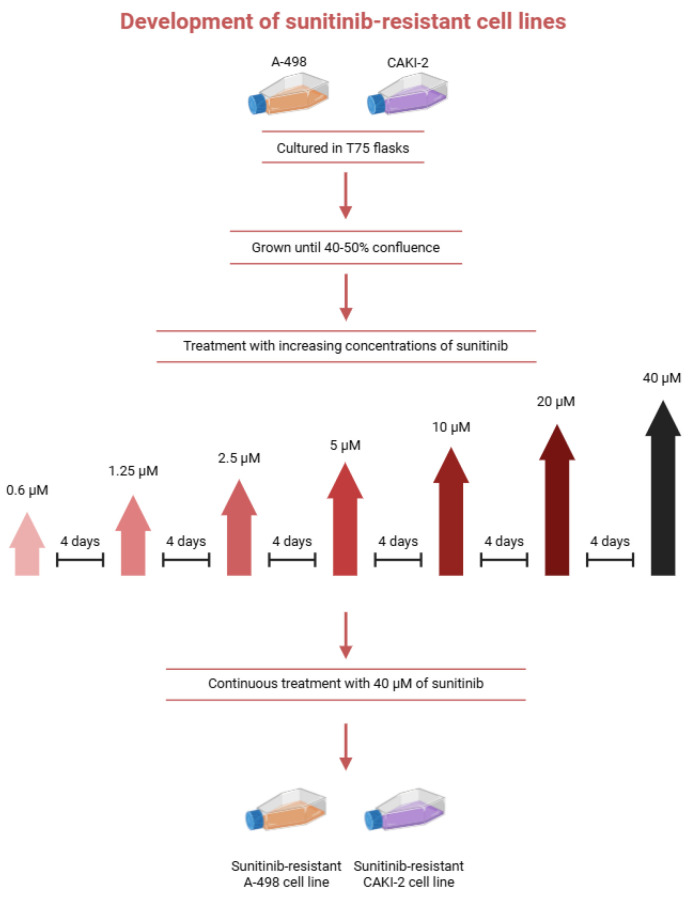
Development of sunitinib-resistant RCC cell lines. Sunitinib was added to the media of A-498 and CAKI-2 cells at increasing concentrations (0.6–40 µM) for 4 weeks. The cell media was changed every 4 days, increasing the concentration of sunitinib each time. After reaching a concentration of 40 µM, the cells were further grown in media containing this exact concentration. Cell viability assay was performed (Figure 2), and IC_50_ values were also determined to demonstrate sunitinib-resistance (Supplementary Figure S1) [29].

To establish sunitinib-resistant cell lines, A-498 and CAKI-2 cells were gradually exposed to increasing concentrations of sunitinib (0.6–40 µM) over a four-week period. Cells were initially seeded in T75 flasks and grown until 40–50% confluence. The media were then replaced with fresh media containing the lowest concentration of sunitinib (0.6 µM). The concentration was increased every four days (1.25 → 2.5 → 5 → 10 → 20 → 40 µM). During the resistance development period, cells were cultured in IMDM supplemented with 10% fetal bovine serum (FBS), 100 U/mL penicillin, and 100 µg/mL streptomycin under the same conditions as the parental cell lines and were regularly passaged to maintain appropriate cell density and morphology. Fresh medium containing the corresponding concentration of sunitinib was replaced every four days according to the selection protocol. During the selection procedure, the response of the developing cell populations to sunitinib was periodically monitored using CellTiter-Blue^®^ cell viability assays (Promega, Madison, WI, USA). Once 40 µM was reached, the resistant cell lines were continuously maintained in medium containing this concentration [29]. The resistant phenotype also was confirmed by CellTiter-Blue^®^ cell viability assays, and IC_50_ values were determined from dose–response curves as described below (Supplementary Figure S1). Figure 1 is a schematic representation of the development of sunitinib-resistant cell lines.

**Figure 2:**
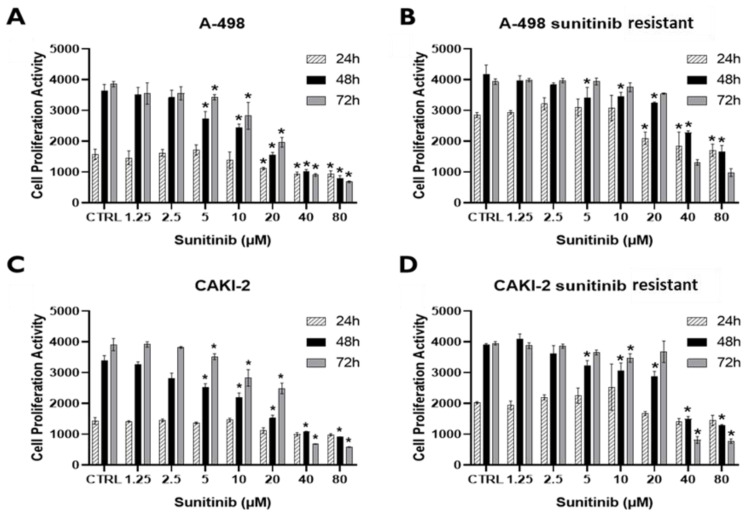
Cell proliferation activity of sunitinib-sensitive and -resistant A-498 (**A**,**B**) and CAKI-2 (**C**,**D**) cell lines. All cell lines were treated with increasing concentrations of sunitinib (1.25–80 µM) for 24, 48, and 72 h. CellTiter-Blue^®^ Cell Viability Assay (Promega, Madison, WI, USA) was performed to detect cell proliferation activity. DMSO was used as a vehicle for untreated control cells (CTRL). * *p* < 0.05 means a significant inhibition of cell growth.

### 2.5. Sunitinib Treatment and Detection of Cell Proliferation Activity

Cell proliferation was assessed using the CellTiter-Blue^®^ Cell Viability Assay. Parental and resistant A-498 and CAKI-2 cells were seeded at 10^4^ cells/well in 96-well plates. After a 24-h pre-incubation in complete medium, the culture medium was replaced with fresh media containing increasing concentrations of sunitinib (1.25–80 µM). The cells were then incubated for 24 to 72 h at 37 °C in a 5% CO_2_ atmosphere. Cell proliferation was measured every 24 h by adding CellTiter-Blue reagent, followed by a 2-h incubation at 37 °C. Fluorescence was measured using a BioTek microplate reader (BioTek, Winooski, VT, USA) at 560/590 nm excitation/emission.

All treated groups were compared to the DMSO-treated control at each time point over the 72-h period. In addition, the half-maximal inhibitory concentration (IC_50_) was determined using GraphPad Prism Nonlinear regression analysis. Concentrations of the drugs used in further cell culture experiments were optimized according to the cell proliferation curves.

### 2.6. Sunitinib Treatment

Based on cell proliferation tests, the IC_50_ value for the resistant CAKI-2 cell line was 31.24 µM, while for the resistant A-498 cell line it was 36.40 µM, after 72 h of sunitinib treatment. Based on these results, for our experiments, both the sensitive and resistant CAKI-2 and A-498 cell lines were treated with sunitinib at a concentration of 40 µM, the dose which resulted in a significant inhibition of cell proliferation in the case of all four cell lines. Sunitinib was added to the media of the cells, and after 24, 48, and 72 h of incubation, total protein was isolated from each cell line. An untreated cell line was used as a control for each sample line. The 40 μM sunitinib concentration used in this study exceeds clinically achievable plasma levels and therefore represents an experimental in vitro resistance model rather than a direct simulation of clinical drug exposure. This concentration was selected based on experimentally determined IC_50_ values and cell proliferation data to ensure measurable biological responses across all investigated cell lines.

### 2.7. Quantitative Real-Time PCR (qRT-PCR)

Total RNA was extracted from both tumorous and adjacent non-tumorous kidney tissues, as well as from untreated cell lines. Isolation was performed using TRIzol Reagent (TR118, Molecular Research Center Inc., Cincinnati, OH, USA). DNase I treatment was carried out for all samples following RNA extraction and before reverse transcription. This step formed part of the RNA isolation procedure, using the rDNase Set supplied by Macherey-Nagel (Cat. No. 10845761, Düren, Germany). RNA concentration and purity were assessed with a NanoDrop ND-1000 spectrophotometer (NanoDrop Technologies, Wilmington, DE, USA). After quality and quantity assessment, the RNA samples were aliquoted and stored at −80 °C until further analysis.

cDNA (complementary DNA) was synthesized from total RNA using the Tetro cDNA Synthesis Kit (Bioline, London, UK), following the manufacturer’s instructions. The final concentration was 1000 ng cDNA per 20 μL. Reverse transcription PCR (RT-PCR) was performed in 20 μL reactions using Oligo (dT)_18_ primers. PCR conditions: 35 cycles of 95 °C for 15 s, 60 °C for 30 s, and 72 °C for 10 s, followed by a final extension at 72 °C for 2 min. Reactions were run on a C1000 Touch Thermal Cycler (Bio-Rad Laboratories Inc., Hercules, CA, USA).

Expression of transporter genes ABCB1, ABCB5, and ABCG2 was measured using iTaq™ Universal SYBR^®^ Green Supermix (Bio-Rad Laboratories, Hercules, CA, USA). Re-actions (10 µL) were run in triplicate on a CFX-96 Real-Time System. Protocol: 10 min at 95 °C, followed by 39 cycles of 95 °C for 15 s and annealing at 57–60 °C for 1 min, depending on primer Tm (Supplementary Table S1).

GAPDH (Glyceraldehyde 3-phosphate dehydrogenase) was used as a reference gene. Expression was calculated using the 2^−ΔΔCT^ method.

### 2.8. Western Blot Analysis

Total protein was isolated from tissue samples and sunitinib-treated cell lines using M-PER lysis buffer (Thermo Fisher Scientific, Waltham, MA, USA), with 30 min incubation on ice and vortexing every 5–10 min. Samples were centrifuged at 14,000× *g* for 5 min at 4 °C. The supernatant was collected and stored at −80 °C. Protein concentration was measured by Bradford assay. A total of 40 µg of protein from each sample was loaded onto the gel. Laemmli buffer and β-mercaptoethanol (9:1) were added, and samples were boiled at 100 °C for 5 min. Proteins were separated via SDS-PAGE (Sodium dodecyl sulfate-polyacrylamide gel electrophoresis) and transferred to PVDF (Polyvinylidene fluoride) membranes (Millipore, Burlington, MA, USA), pre-activated with methanol. Membranes were incubated overnight at 4 °C with primary antibodies (listed in Supplementary Table S2), washed with TBST (Tris buffered saline with Tween-20), then incubated for 90 min with secondary antibodies at room temperature. Chemiluminescent detection was used, and signals were recorded with a ChemiDoc Touch imaging system (Bio-Rad Laboratories, Hercules, CA, USA). To control for equal loading, protein signals were normalized to the corresponding housekeeping protein, hypoxanthine phosphoribosyltransferase (HPRT; Boster Biological Technology, Pleasanton, CA, USA), Akt (Cell Signaling Technology, Danvers, MA, USA), or extracellular signal-regulated kinase (ERK; Cell Signaling Technology, Danvers, MA, USA). For ERK signaling analysis, total ERK protein levels were first normalized to the HPRT loading control, after which phosphorylated ERK levels were normalized to the corresponding total ERK protein to determine ERK activation. Band intensities were quantified using Image Lab software (version 5.2.1), and the resulting normalized values were exported for downstream analysis. All measurements were obtained from three independent biological replicates (*N* = 3). Densitometric analyses were performed using these three independent biological replicates and the results were normalized to the corresponding loading control.

### 2.9. Statistical Analysis

For qRT-PCR experiments, technical replicates were used three times (*n* = 3), whereas the Western blot experiments were conducted with three independent biological replicates (*N* = 3), to ensure statistical accuracy in both cases. GraphPad Prism 5.0 (GraphPad Software Inc., San Diego, CA, USA) was used for statistical analysis via either multiple unpaired *t*-test or two-way ANOVA, followed by Sidak’s multiple comparison test. Differences with a *p*-value ≤ 0.05 were considered statistically significant. qRT-PCR data are expressed as fold change ± standard error of the mean (±SEM) from three technical replicates. Western blot data are shown as the mean ± standard error (±SEM).

## 3. Results

### 3.1. Analysis of Human Samples

Histopathological examination was carried out to confirm the presence of cancer, with only a minimal amount of non-malignant tissue present. With the exception of two tissue samples, all the samples studied were originated from primary surgical renal cancers. Tumors were staged according to the TNM staging system. Out of the 20 patients, two with metastases received systemic drug treatment, one of whom was treated with sunitinib. Only two patients received chemotherapy or hormone therapy due to the presence of a concomitant tumor, and one patient underwent radiation therapy. In the remaining cases, systemic therapy was either not administered or data regarding treatment was unavailable (Table 1).

### 3.2. Examination of Transporter Genes ABCB1, ABCB5, and ABCG2 in Human Renal Cancer

**Figure 3:**
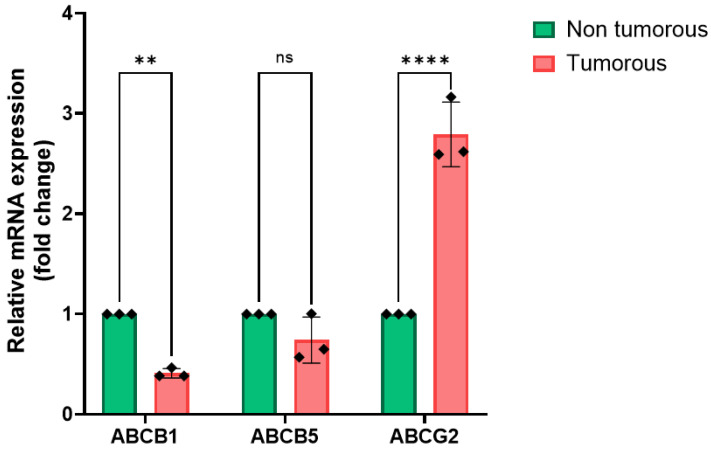
Comparison of the expression of ABC transporter genes in the studied human kidney tissue sample pairs. The relative mRNA expressions of ABCB1, ABCB5, and ABCG2 transporter genes were examined in 20 tumorous and adjacent non-tumorous tissue samples with qRT-PCR technique. The experiment was performed in three technical replicates (*n* = 3). GAPDH was used as an endogenous control housekeeping gene to normalize the relative mRNA expression values of the transporter genes. Relative expression levels in tumorous samples were normalized to their adjacent non-tumorous controls using the 2^−ΔΔCT^ method. Data is represented as fold change ± standard error of the mean (±SEM). Two-Way ANOVA with Sidak’s multiple comparison test was used to detect significant differences (** *p* < 0.01; **** *p* < 0.0001; ns = not significant). Primer sequences used in the study are listed in Table S1.

**Figure 4:**
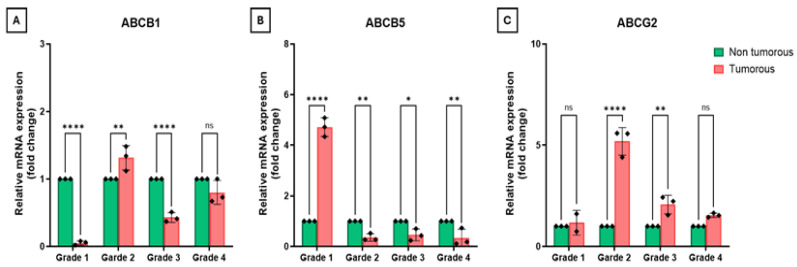
Expression of (**A**) ABCB1, (**B**) ABCB5, and (**C**) ABCG2 genes in relation to pathological grade. GAPDH was used as an endogenous control housekeeping gene to normalize the relative mRNA expression values of the transporter genes. Relative expression levels in tumorous samples were normalized to their adjacent non-tumorous controls using the 2^−ΔΔCT^ method. The experiment was performed in three technical replicates (*n* = 3). Data is represented as fold change ± standard error of the mean (±SEM). Two-way ANOVA with Sidak’s multiple comparison test was used to detect significant differences (* *p* < 0.05; ** *p* < 0.01; **** *p* < 0.0001; ns = not significant). Primer sequences are listed in Table S1.

To investigate the expression of ABC transporters, we first examined the gene ex-pression levels of ABCB1, ABCB5, and ABCG2 in 20 paired non-tumorous and tumorous human kidney tissue samples obtained from the Department of Urology at the University of Debrecen. qRT-PCR analysis of these paired samples is presented in Figure 3. Based on the known clinicopathological characteristics of the patients, we also analyzed the expression of the studied transporter genes at mRNA level based on the pathological grades. The results of this correlation analysis are also shown in Figure 4.

Based on our data, ABCB1 showed consistently higher levels in non-tumorous samples compared to tumorous ones (Figure 3). To better understand this difference, we analyzed expression patterns across pathological grades. In Grades 1, 3 and 4, ABCB1 ex-pression remained higher in non-tumorous tissues; however, in Grade 2, ABCB1 expression became higher in tumorous samples (Figure 4A). Interestingly, ABCB5 expression was also higher in non-tumorous tissues overall (Figure 3). However, the grade-specific data were inconsistent: expression was significantly higher in tumorous samples in Grade 1, but elevated in non-tumorous tissues in Grades 2, 3 and 4 (Figure 4B). Out of the three transporter genes examined, ABCG2 showed the biggest difference in expression levels, reaching an almost three-fold change in tumorous samples, compared to the non-tumorous ones (Figure 3). ABCG2 expression remained higher in tumorous samples, regardless of pathological grade (Figure 4C).

### 3.3. Effect of Treatment with Sunitinib on Cell Proliferation Activity on the Studied Sunitinib-Sensitive and Sunitinib-Resistant CAKI-2 and A-498 Cell Lines

Cell proliferation activity after sunitinib treatment of sunitinib-sensitive and sunitinib-resistant CAKI-2 and A-498 cell lines is presented in Figure 2.

Sunitinib inhibited cell proliferation in both parental and resistant cell lines in a dose-dependent manner. A significant antiproliferative effect was observed at 20 µM in the parental A-498 and CAKI-2 cells, whereas the resistant counterparts required 40 µM to achieve a comparable response, confirming their resistant phenotype (Figure 2). Distinct morphological differences between parental and resistant cells were also detected using an inverted microscope (Nikon Instruments Inc., Melville, NY, USA).

### 3.4. Determination of IC_50_ Values and Application in Further Experiments

IC_50_ values of sunitinib were determined by cell-proliferation assays in all four cell lines (sensitive and resistant CAKI-2 and A-498) after 72 h of treatment.

Dose–response analyses demonstrated a clear concentration- and time-dependent decrease in cell viability in both sensitive and sunitinib-resistant CAKI-2 and A-498 cell lines following sunitinib treatment. Increasing incubation time resulted in a marked left-ward shift of the dose–response curves in all cell lines, indicating enhanced drug efficacy at longer exposure times.

In the sensitive CAKI-2 cell line, the IC_50_ values decreased substantially from 151.9 µM at 24 h to 22.31 µM at 48 h and 21.73 µM at 72 h. In contrast, the sunitinib-resistant CAKI-2 cells exhibited consistently higher IC_50_ values at all time points (122.7 µM at 24 h, 36.06 µM at 48 h and 31.24 µM at 72 h), confirming reduced sensitivity to sunitinib (Supplementary Figure S1).

Similarly, in the sensitive A-498 cell line, IC_50_ values decreased from 92.67 µM at 24 h to 20.22 µM at 48 h and remained comparable at 72 h (20.09 µM). The sunitinib-resistant A-498 cells showed markedly higher IC_50_ values (85.11 µM at 24 h, 54.93 µM at 48 h and 36.40 µM at 72 h), indicating a persistent resistant phenotype (Supplementary Figure S1).

Overall, both sunitinib-resistant CAKI-2 and A-498 cell lines displayed reduced responsiveness to sunitinib compared with their sensitive counterparts, particularly at 48 and 72 h. Based on the IC_50_ values determined for all four cell lines at 72 h, a treatment concentration of 40 µM sunitinib was selected for subsequent experiments. The 40 µM sunitinib concentration mainly was used to create an in vitro resistance model and does not reflect clinical plasma levels.

### 3.5. Examination of the Expression of ABC Transporters in Human Kidney Cancer Cell Lines CAKI-2 and A-498

Based on the results of the cell proliferation activity after sunitinib treatment of sunitinib-sensitive and sunitinib-resistant CAKI-2 and A-498 cell lines, and also on the calculated IC_50_ values, 40 µM sunitinib was selected for all subsequent experiments in both sensitive and resistant A-498 and CAKI-2 cell lines. Cells were treated in parallel and analyzed after 24, 48, and 72 h, with an untreated group included for each cell line as the control. Treated cells were subsequently used for further analyses.

**Figure 5:**
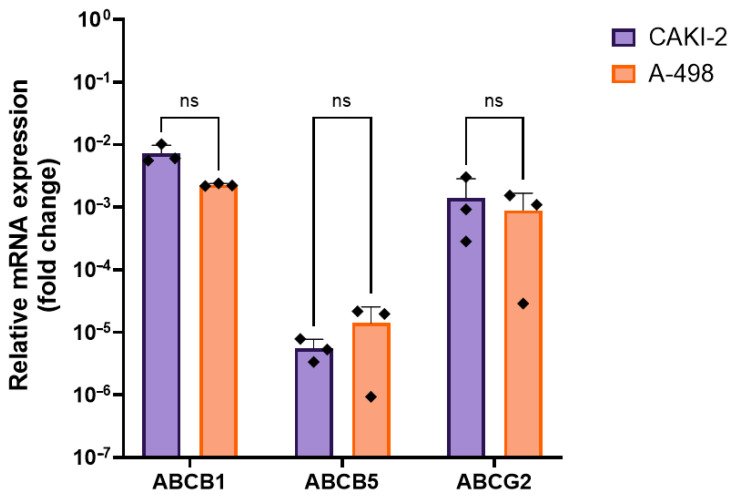
The expressions of the ABCB1, ABCB5 and ABCG2 transporter genes were examined in untreated CAKI-2 and A-498 human RCC cell lines, to show the differences regarding gene expression between the two cell lines. Total RNA was isolated from each cell lines, and the experiment was performed in three technical replicates (*n* = 3). Expression values were normalized to GAPDH, which was used as an endogenous control. Relative expression values were calculated using the 2^−ΔCT^ method. Data is represented as fold change ± standard error of the mean (±SEM). Multiple unpaired *t*-test was used to detect significant differences (* *p* < 0.05; ns = not significant). Primer sequences are listed in Table S1.

Next, we examined the baseline expression levels of the ABC transporter genes in untreated cells. As shown in Figure 5, ABCB1 and ABCG2 showed a higher expression in the CAKI-2 cell line when compared to the A-498 cell line, while ABCB5 showed higher expression in the A-498 cells when compared to the CAKI-2 cell line. However, it is important to highlight that none of these differences were statistically significant.

**Figure 6:**
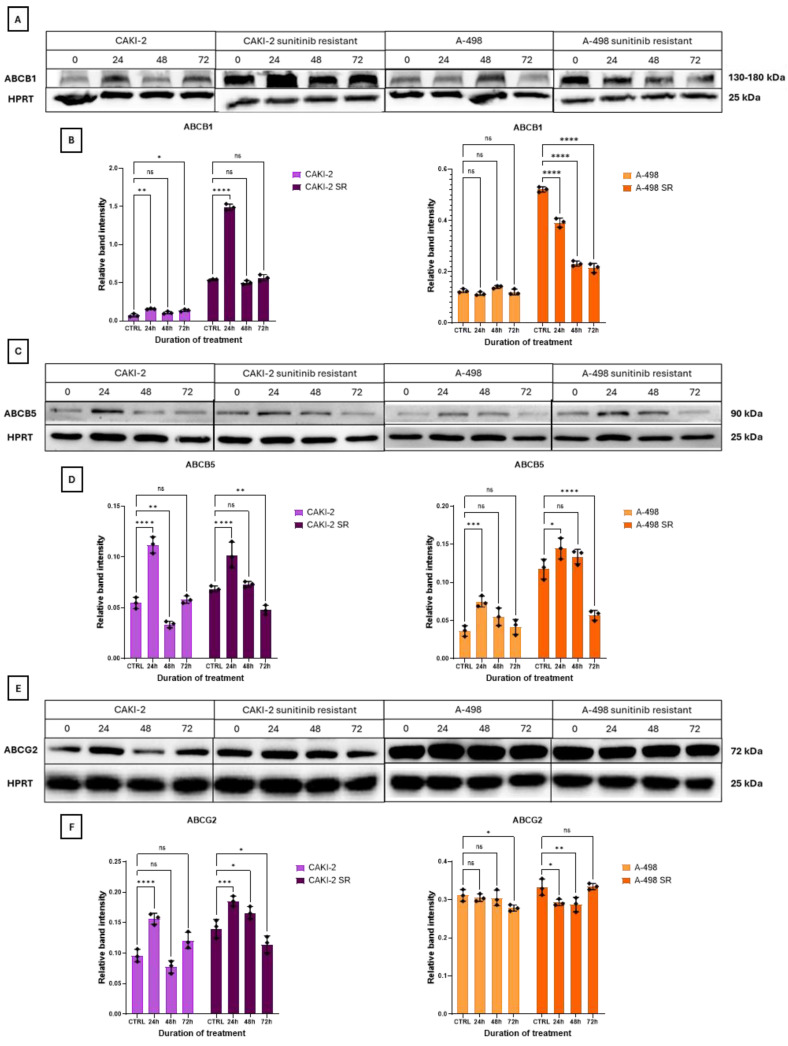
Western blot analysis of ABC transporter protein expression after 40 µM sunitinib treatment. The expression of ABCB1 (**A**,**B**), ABCB5 (**C**,**D**), and ABCG2 (**E**,**F**) transporter proteins were examined in both sunitinib-sensitive and resistant CAKI-2 and A-498 human RCC cell lines after 24, 48, and 72 h of sunitinib treatment. Untreated cell lines were used as control groups. Expression values were normalized to HPRT as an endogenous control. Band intensities were quantified using Image Lab software (Bio-Rad Laboratories, Hercules, CA, USA). Representative Western blot images from one of three independent biological experiments are shown. Data from three distinct experiments (N = 3) is represented as the mean ± standard error (±SEM). Two-way ANOVA with Sidak’s multiple comparison test was used for the statistical analysis (* *p* < 0.05; ** *p* < 0.01; *** *p* < 0.001; **** *p* < 0.0001; ns = not significant).

As described above, the 40 µM sunitinib concentration was selected based on the experimentally determined IC_50_ values and cell proliferation assays and was used throughout the subsequent experiments. (Supplementary Figure S1). The proteins of interest were isolated from 40 µM sunitinib-treated, sunitinib-sensitive and -resistant CAKI-2 and A-498 cell lines. The results of the Western blot measurements are shown in Figure 6.

Western blot analysis showed that the expression of the ABCB1 protein was in-creased in the sensitive CAKI-2 cell line after 40 μM sunitinib treatment compared to the control, then slightly declined at 48 h, followed by another increase at 72 h. Expression remained above control levels throughout. In the resistant CAKI-2 line, the protein level increased after 24 h, then decreased at 48 h. At 72 h, the protein level returned close to the control level (Figure 6B). In the sensitive A-498 line, similar changes have occurred as in the sensitive CAKI-2 cell line; however, a slight increase was observed at 48 h. In the resistant A-498 line, ABCB1 expression continuously decreased after treatment, remaining below the control level at all time points. Overall, compared with the corresponding sensitive cell lines, the resistant CAKI-2 and A-498 cells generally exhibited higher ABCB1 protein expression during sunitinib treatment (Figure 6B).

ABCB5 protein expression in sensitive CAKI-2 cells increased significantly after 24 h of sunitinib treatment, decreased at 48 h, and increased again at 72 h. In resistant CAKI-2 cells, ABCB5 levels were elevated at 24 h but subsequently declined at 48 and 72 h (Figure 6D). In sensitive A-498 cells, ABCB5 expression increased at 24 h and decreased at 48 and 72 h, whereas resistant A-498 cells exhibited increased expression at 24 and 48 h followed by a decrease at 72 h (Figure 6D). In contrast to ABCB1, ABCB5 expression displayed a more dynamic and cell-line-dependent pattern. While differences between sensitive and resistant cells were observed, no consistent trend was detected throughout the treatment period, suggesting that ABCB5 regulation may be influenced by additional cellular factors.

ABCG2 protein levels in sensitive CAKI-2 cells significantly increased at 24 h, dropped at 48 h, then elevated again above control at 72 h. In the resistant CAKI-2 line, expression increased after 24 h, then decreased below control over the following two days (Figure 6F). In the sensitive A-498 line, no major changes were detected at 24 or 48 h, though expression slightly dropped at 72 h. In the resistant line, protein levels decreased after 24 h and remained low at 48 h but increased by 72 h to near-control levels (Figure 6F). Compared with the corresponding sensitive cell lines, ABCG2 expression in resistant cells showed a variable temporal pattern without a uniform increase throughout treatment, indicating that its regulation during acquired sunitinib resistance may be more complex than that of ABCB1.

### 3.6. Analysis of the Signaling Mechanisms Behind Sunitinib Resistance

We also studied the possible signaling pathways that regulate apoptosis and cell proliferation. We examined the effect of higher doses of sunitinib treatment on the expression of these proteins.

One of the well-examined signaling pathways is the PI3K/Akt/mTOR (Phosphoinositide 3-kinase/Protein kinase B/Mammalian target of rapamycin) pathway. It is important to note that many TKIs target the kinase components of this pathway [24]; therefore, the activation of PI3K/Akt/mTOR signaling might correlate with sunitinib resistance.

**Figure 7:**
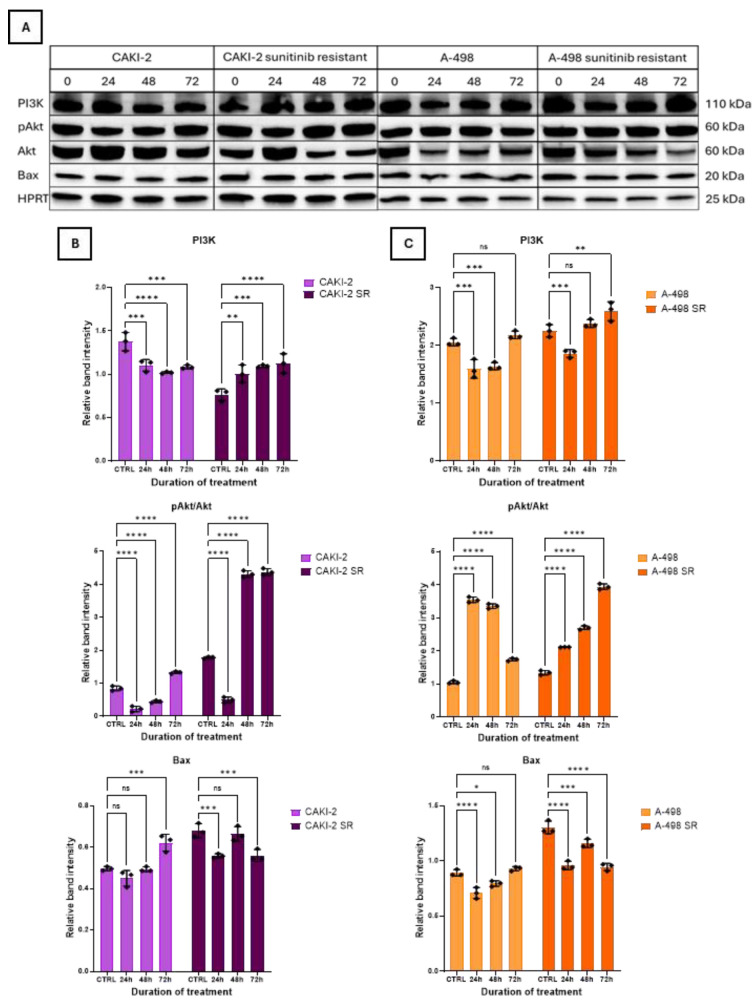
Western Blot analysis of protein expression after 40 µM sunitinib treatment. Expression changes of PI3K, pAkt/Akt, and Bax proteins were examined after 24, 48, and 72 h of sunitinib treatment. (**A**) Western blot images for PI3K, pAkt/Akt, and Bax protein expression. (**B**) Band intensities for sunitinib-sensitive and -resistant CAKI-2 cell lines. (**C**) Band intensities for sunitinib-sensitive and -resistant A-498 cell lines. Untreated cells were used as control groups, and the intensities of the sunitinib treated samples were normalized to HPRT or Akt. Band intensities were quantified using Image Lab software (Bio-Rad Laboratories, Hercules, CA, USA). Representative Western blot images from one of three independent biological experiments are shown. Data from three distinct experiments (N = 3) is represented as the mean ± standard error (±SEM). Two-way ANOVA with Sidak’s multiple comparison test was used for the statistical analysis (* *p* < 0.05; ** *p* < 0.01; *** *p* < 0.001; **** *p* < 0.0001; ns = not significant).

In the sensitive CAKI-2 cell line, the expression of PI3K decreased 24 h after treatment, then increased again after 72 h. In contrast, in the resistant cell line, it was increased above the control level after 24 h and remained above control level at all times (Figure 7B). In the sensitive A-498 cell line, protein expression decreased significantly after 24 h, then began to increase continuously. In the resistant cell line, similar changes were observed (Figure 7C).

The expression of the pAkt (phospho-Akt) protein in the sensitive CAKI-2 cell line decreased significantly after 24 h, then started to increase continuously over the next 2 days. In the resistant cell line, a significant decrease was also observed after 24 h, followed by a significant increase after 48 and 72 h. (Figure 7B). In the sensitive A-498 cell line, a significant increase was observed in pAkt protein expression during the first two days, followed by a certain decrease on the third day; however, protein levels remained above the control level at all times. In the resistant cell line, pAkt levels significantly increased 24 h after sunitinib treatment, and this trend continued over the following two days (Figure 7C).

Sunitinib treatment resulted in an increase of Bax (Bcl-2-like protein 4) protein levels in the sensitive CAKI-2 and A-498 renal cancer cell lines, whereas in the resistant counterparts, Bax expression initially exhibited a modest rise at 48 h, followed by an overall decline, suggesting a differential apoptotic response associated with treatment sensitivity (Figure 7B,C).

Overall, resistant cell lines demonstrated a different temporal activation pattern of the PI3K/Akt pathway compared with the sensitive cell lines, suggesting altered signaling dynamics associated with the resistant phenotype.

### 3.7. Analysis of PTEN Expression After Sunitinib Treatment

**Figure 8:**
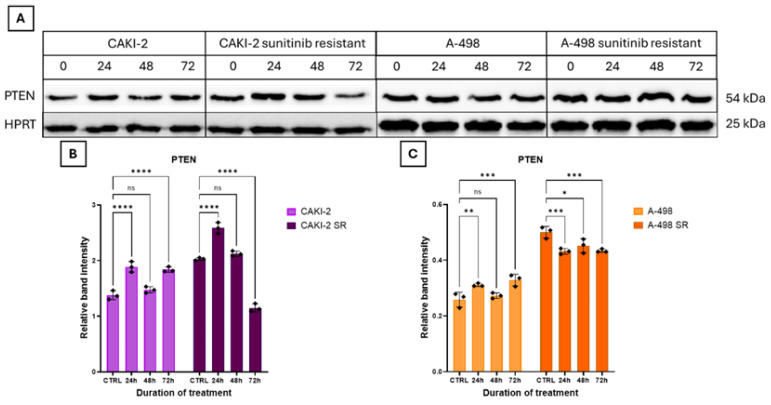
Western Blot analysis of protein expression after 40 µM sunitinib treatment. Expression changes of PTEN were examined after 24, 48, and 72 h of sunitinib treatment. (**A**) Western blot images for PTEN protein expression. (**B**) Band intensities for sunitinib-sensitive and -resistant CAKI-2 cell lines. (**C**) Band intensities for sunitinib-sensitive and -resistant A-498 cell lines. Untreated cells were used as control groups, and the intensities of the sunitinib treated samples were normalized to HPRT. Band intensities were quantified using Image Lab software (Bio-Rad Laboratories, Hercules, CA, USA). Representative Western blot images from one of three independent biological experiments are shown. Data from three distinct experiments (*N* = 3) is represented as the mean ± standard error (±SEM). Two-way ANOVA with Sidak’s multiple comparison test was used for the statistical analysis (* *p* < 0.05; ** *p* < 0.01; *** *p* < 0.001; **** *p* < 0.0001; ns = not significant).

PTEN (phosphatidylinositol-3,4,5-trisphosphate 3-phosphatase) protein expression in the sensitive CAKI-2 cell line fluctuated continuously during the treatment period. In the resistant CAKI-2 cell line, expression increased 24 h after sunitinib treatment, then began to decrease continuously. After 72 h, PTEN expression decreased below the control level (Figure 8B). In the sensitive A-498 cell line, a significant change was observed only after 72 h, when expression significantly increased. In contrast, in the resistant A-498 cell line, protein expression decreased significantly after 24 h and remained at approximately the same level throughout the experiment (Figure 8C).

### 3.8. Analysis of NF-κB Expression After Sunitinib Treatment

**Figure 9:**
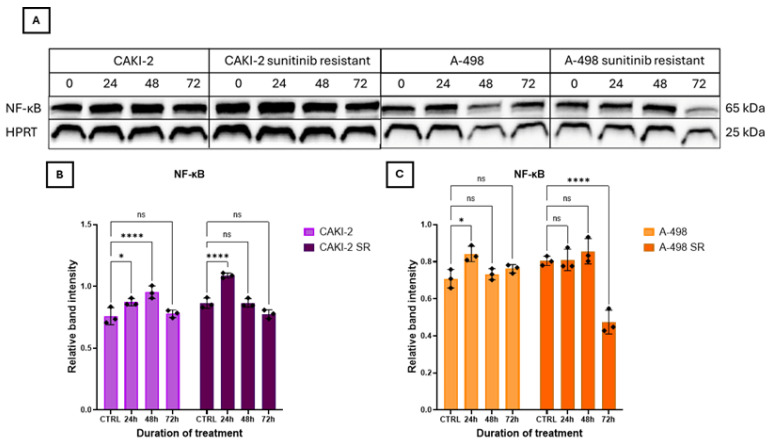
Western Blot analysis of protein expression after 40 µM sunitinib treatment. Expression changes of NF-κB were examined after 24, 48, and 72 h of sunitinib treatment. (**A**) Western blot images for NF-κB protein expression. (**B**) Band intensities for sunitinib-sensitive and -resistant CAKI-2 cell lines. (**C**) Band intensities for sunitinib-sensitive and -resistant A-498 cell lines. Untreated cells were used as control groups, and the intensities of the sunitinib treated samples were normalized to HPRT. Band intensities were quantified using Image Lab software (Bio-Rad Laboratories, Hercules, CA, USA). Representative Western blot images from one of three independent biological experiments are shown. Data from three distinct experiments (*N* = 3) is represented as the mean ± standard error (±SEM). Two-way ANOVA with Sidak’s multiple comparison test was used for the statistical analysis (* *p* < 0.05; **** *p* < 0.0001; ns = not significant).

NF-κB (Nuclear factor kappa-light-chain-enhancer of activated B cells) protein expression in the sensitive CAKI-2 cell line increased continuously for 48 h, then decreased reaching control levels 72 h after sunitinib treatment. In the resistant cell line, significant changes were observed only after 24 h of treatment (Figure 9B). In the sensitive A-498 cell line, protein expression was fluctuating throughout the experiment. However, in the resistant cell line, there was a significant decrease in protein expression on the third day after treatment (Figure 9C). Compared with sensitive cells, resistant cell lines generally exhibited sustained NF-κB activation during sunitinib treatment, although the magnitude and timing differed between the two RCC models.

### 3.9. Analysis of pERK/ERK Expression After Sunitinib Treatment

**Figure 10:**
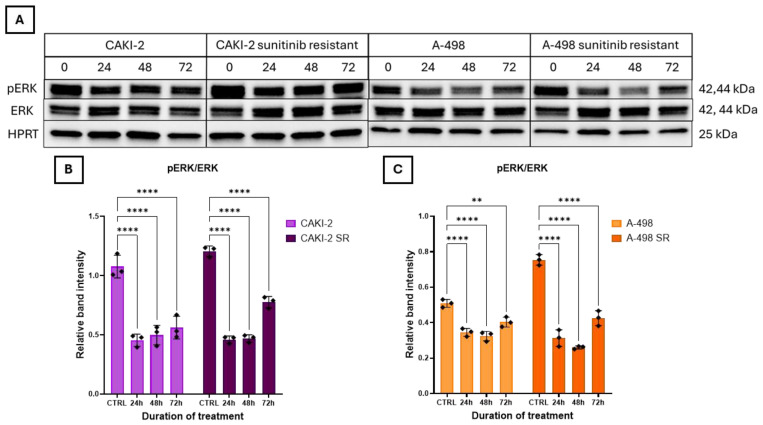
Western Blot analysis of protein expression after 40 µM sunitinib treatment. Expression changes of pERK/ERK proteins were examined after 24, 48, and 72 h of sunitinib treatment. (**A**) Western blot images for pERK and ERK protein expression. (**B**) Band intensities for sunitinib-sensitive and -resistant CAKI-2 cell lines. (**C**) Band intensities for sunitinib-sensitive and -resistant A-498 cell lines. Untreated cells were used as control groups, and the intensities of the sunitinib treated samples were normalized to ERK. Total ERK protein levels were first normalized to the HPRT. Band intensities were quantified using Image Lab software (Bio-Rad Laboratories, Hercules, CA, USA). Representative Western blot images from one of three independent biological experiments are shown. Data from three distinct experiments (*N* = 3) is represented as the mean ± standard error (±SEM). Two-way ANOVA with Sidak’s multiple comparison test was used for the statistical analysis (** *p* < 0.01; **** *p* < 0.0001; ns = not significant).

In both the sensitive and resistant CAKI-2 cell lines, the expression level of the pERK (Phospho-ERK) protein decreased significantly after sunitinib treatment, then started to increase continuously (Figure 10B). In the sensitive A-498 cell line, a significant decrease was observed after 24 h. However, by 72 h, the expression level slightly increased again. In the resistant A-498 cell line, expression of the examined protein was significantly reduced following sunitinib treatment compared to the control, and this downregulated level remained stable throughout the treatment period (Figure 10C). Differences in ERK activation kinetics were observed between sensitive and resistant cells, indicating altered MAPK signaling during the development of sunitinib resistance.

## 4. Discussion

Renal cell carcinoma, particularly the clear-cell subtype, often exhibits resistance to tyrosine kinase inhibitors such as sunitinib, limiting treatment efficacy and patient outcomes. ATP-binding cassette transporters, including ABCB1, ABCB5 and ABCG2, are known to mediate drug efflux, contributing to multidrug resistance and impacting TKI bioavailability [12,17,30,31,32,33]. Sunitinib, a multitargeted tyrosine kinase inhibitor, is a cornerstone in treating metastatic renal cell carcinoma. Its effectiveness is influenced by ABC transporters—primarily ABCB1, ABCG2, and ABCB5—which regulate drug efflux and contribute to resistance [10,33,34,35]. While ABCB5’s role in resistance is less defined, emerging evidence indicates it may contribute to multidrug resistance across cancers, including RCC [5,25].

Understanding the expression patterns of these transporters in RCC may aid the development of personalized therapies to overcome resistance and improve clinical out-comes [36,37]. This study investigates the expression of ABCB1, ABCB5, and ABCG2 in human renal tumors to better inform targeted treatment strategies.

In paired tumorous and adjacent non-tumorous human kidney tissues, ABCB1 showed the highest overall expression, with consistently higher levels in non-tumorous tissues in most of the pathological grades. This aligns with earlier findings that ABCB1 plays an essential physiological role in proximal renal tubules [18]. Contrary to ABCB1, ABCG2 exhibited generally higher expression levels in tumorous tissues, regardless of pathological grade. This may reflect tumor-specific adaptation mechanisms enhancing drug resistance. ABCG2 is known to transport TKIs such as sunitinib [9]. ABCB5 expression was generally lower in tumorous tissues compared to non-tumorous tissues, except in G1 tumors, suggesting a complex and potentially a grade-dependent regulation of that ABCB5 expression in RCC [14]. Our data of ABC transporter analyses at mRNA level further revealed inter-individual variability and co-expression among the three genes, rein-forcing the potential clinical relevance of transporter profiling despite the relatively small sample size (20–20 paired tumorous and non-tumorous samples).

Our analysis of CAKI-2 and A-498 ccRCC cell lines complemented the tissue findings, revealing complex, cell-line-specific regulation in association with sunitinib-resistance. Baseline expression showed higher ABCB1 and ABCG2 levels in CAKI-2 cells, consistent with the established role of these transporters in drug efflux and sunitinib resistance [34,38]. After 40 µM sunitinib treatment, resistant lines exhibited persistently elevated ABCB1 expression compared to the sensitive cell, indicating an association between ABCB1 expression and the resistant phenotypes [39]. ABCG2 displayed fluctuating protein levels after the sunitinib treatment, aligning with its earlier tissue overexpression [40,41]. The difference between ABCB5 mRNA expression in tissue samples and protein expression in resistant cell lines suggests that post-transcriptional regulatory mechanisms may contribute to the regulation of ABCB5 expression [42]. Taken together, these findings underscore the heterogeneity of transporter regulation and the need to assess both transcript and protein levels for therapeutic insight [43,44]. Overall, resistant RCC cell lines displayed expression patterns that differed from those of their sensitive counterparts throughout sunitinib treatment. In particular, ABCB1 expression generally remained higher in resistant cells, whereas ABCB5 and ABCG2 exhibited more dynamic and cell-line-dependent changes.

The differences observed between the clinical specimens and the resistant cell models most likely reflect the differences between untreated primary tumors and the experimental cell model used in this study. The relatively small and heterogeneous patient cohort may have further contributed to the variability in transporter expression.

Our combined human tissue specimens and experimental cell line data support the association of ABC transporters, particularly ABCB1, with major signaling pathways, including PI3K/Akt/mTOR, MAPK/ERK, and NF-κB. For instance, previous studies have suggested that activation of NF-κB may suppress p53-mediated apoptosis and drive transporter upregulation, thereby promoting survival in resistant cells. Preclinical evidence shows that sunitinib is a substrate of ABCB1 and ABCG2 and that combined inhibition of transporters can enhance intracellular drug accumulation which might lead to drug resistance [30,45,46]. Meta-analysis data highlight that genetic variants in ABCG2 and ABCB1 can predict toxicities and progression-free survival in mRCC patients [34]. These insights suggest that molecular profiling of transporter and signaling pathway statuses may support personalized sunitinib therapy and guide combination strategies with transporter inhibitors or pathway modulators. According to our results the ABC transporters studied might differently interact with each other in the studied sunitinib-sensitive and -resistant CAKI-2 and A-498 human kidney cancer cell lines. The cell-type-dependent interaction of ABC transporters suggests their specific role in personalized therapy of the patients.

The interplay between key signaling pathways and ABC transporters, specifically ABCB1, ABCB5, and ABCG2, might have a crucial role in modulating drug resistance in renal cell carcinoma. These transporters are not only responsible for drug efflux but are also regulated by signaling cascades such as PI3K/Akt and MAPK/ERK, which influence their expression and function in response to sunitinib treatment [47,48]. ATP-binding cassette transporters might play a critical role in modulating sunitinib efficacy and resistance in RCC. In our study, we examined the expression of key ABC transporters—ABCB1, ABCB5, and ABCG2—in sunitinib-sensitive and -resistant CAKI-2 and A-498 RCC cell lines. While ABCB5 and ABCG2 showed comparable expression levels in both sensitive and resistant lines, ABCB1 was markedly upregulated in both resistant cell lines. This finding aligns with existing literature implicating ABCB1 in multidrug resistance across several cancer types, though its role in RCC remains less defined [49].

Treatment with 40 μM sunitinib led to an initial increase in ABCB1 expression in the resistant CAKI-2 cells. While this upregulation was followed by a significant decrease after 48 h, protein level returned back to the control level by 72 h, suggesting an adaptive cellular response that may be associated with the resistant phenotype. Although in the literature sunitinib has been reported to inhibit ABCB1 activity, transporter activity and their expression at mRNA and protein levels are distinct processes. Therefore, the delayed increase in ABCB1 expression may represent an adaptive response to prolonged drug-induced stress, potentially associated with lysosomal sequestration of sunitinib [49,50,51,52]. In contrast, ABCB5 was only upregulated in the resistant A-498 cell line, consistent with its less established role in sunitinib resistance. Conversely, ABCG2 expression consistently decreased in both cell lines throughout treatment, suggesting that under these experimental conditions it may not be a major contributor to the observed resistant phenotypes [53]. Since ABCG2 was significantly overexpressed in tumorous tissue samples, it would have been reasonable to assume that this transporter plays a dominant role in resistance; however, our results indicate stronger association of ABCB1 expression with the resistant phenotypes than that observed for ABCG2 under the experimental conditions used in this study [49,53].

Molecular pathway analysis highlighted the involvement of PI3K/Akt/mTOR and MAPK/ERK signaling in sunitinib response [24,54]. Our results demonstrated a significant increase in PI3K and pAkt protein expression in resistant cell lines, suggesting that activation of the PI3K/Akt pathway could be associated with the resistant phenotypes. The primary role of this pathway is to promote cell survival, but its excessive activation is strongly associated with drug resistance [54,55]. Based on literature data a reduction in total AKT and mTOR expression could be accompanied by increased PTEN levels, suggesting complex regulatory interplay governing cell survival and apoptosis. PTEN expression exhibited dynamic and cell-line-dependent changes rather than a consistent increase during sunitinib treatment. While pAkt levels progressively increased in both resistant cell lines, total PTEN protein expression fluctuated, suggesting that Akt activation is likely regulated by multiple mechanisms what could be beyond PTEN abundance alone. Since total PTEN levels do not necessarily reflect phosphatase activity, we might suggest that post-translational modifications, including phosphorylation, may contribute to the observed signaling pattern. Of course, further studies investigating PTEN activity and phosphorylation will be required to clarify the molecular mechanisms underlying this response [55,56,57].

The relatively high PTEN expression observed in resistant cells may also explain why NF-κB expression showed only minimal differences between sensitive and resistant cell lines, despite literature evidence linking PI3K/Akt activation to NF-κB–mediated ABCB1 upregulation. These findings may indicate that additional regulatory mechanisms beyond NF-κB might contribute to ABCB1 expression in RCC cells. In contrast, our findings showed a slight decrease in Bax levels in resistant cell lines, whereas sensitive cells exhibited an increasing trend. Given that Bax activation is a key component of sunitinib-induced apoptosis, this reduction may reflect the reduced sensitivity of the resistant cell models to sunitinib [53,54,55,56,57,58,59]. Although NF-κB expression levels showed only minimal differences between sensitive and resistant cell lines, functional differences in pathway activity may still contribute to survival signaling and resistance mechanisms [59,60]. These findings are consistent with the known upregulation of HIF-1α and NF-κB due to VHL mutations in clear cell RCC, which amplify VEGF-driven angiogenesis and resistance [61,62,63].

The MAPK/ERK pathway, another critical regulator of tumor cell survival, often acts in parallel with the PI3K/Akt pathway, although resistance can develop independently through each pathway. In our study, TKI treatment resulted in a marked decrease in pERK levels, consistent with previous findings. This reduction may explain the relatively low ABCB5 expression observed in resistant cells, as earlier studies have demonstrated a direct correlation between MAPK/ERK activity and ABCB5 expression [64,65,66].

Overexpression of ABC transporters has been associated with reduced intracellular drug accumulation in multidrug-resistant cancer cells and may also influence the tumor immune microenvironment. Since cytokines can regulate ABC transporter expression, these interactions may contribute to altered drug response and the development of sunitinib resistance. Targeting ABC transporters may boost the efficacy of immune checkpoint therapies [64,65]. Sunitinib has been shown to inhibit the efflux activity of ABCB1 and ABCG2, enhancing intracellular drug accumulation [66]. Additionally, the relatively low ABCB5 expression may be related to the simultaneous overexpression of ABCB1 and ABCG2 transporters. Their dominant activity could render further ABCB5 upregulation redundant, particularly in the context of PI3K/Akt pathway activation, which is known to strongly regulate ABCB1 and ABCG2 but may have a lesser effect on ABCB5. This interplay underscores the complexity of sunitinib pharmacodynamics in RCC [67,68,69].

In summary, our findings demonstrate associations between altered ABC transporter expression, particularly ABCB1, and changes in PI3K/Akt, MAPK/ERK, and NF-κB signaling pathways in the studied experimental CAKI-2 and A-498 cell lines of sunitinib resistance [68,69,70]. However, the present study does not establish direct mechanistic relationships between these events, further functional validation will be required to establish their direct mechanistic roles. Understanding these molecular mechanisms may guide the development of combinatorial therapeutic strategies targeting both transporter function and associated pathways to enhance treatment efficacy in RCC [70].

## 5. Conclusions

Our findings highlight the potential involvement of ABC transporters—particularly ABCB1, ABCB5, and ABCG2—in mediating sunitinib resistance in renal cell carcinoma. Their dynamic regulation across tumor tissues and cell lines, together with their interaction with key signaling pathways, suggests a multifactorial resistance mechanism. Integrating transporter profiling with pathway analysis may support future efforts aimed at identifying patients at risk for poor response. These insights highlight the need for personalized therapeutic strategies and further clinical exploration of combination treatments targeting both transporters and oncogenic signaling axes. Our findings demonstrate distinct expression patterns of ABCB1, ABCB5, and ABCG2 in RCC tissues and experimental sunitinib-resistant cell models. These transporters may be associated with sunitinib resistance and altered signaling pathways in RCC; however, further functional studies are required to clarify their mechanistic roles.

## 6. Limitations

This study is somehow limited by a relatively small sample size and incomplete clinical data for some cases, which may affect the applicability of the findings. Further research with larger cohorts and comprehensive clinical information is needed to validate and expand upon these results. Furthermore, a comprehensive elucidation of the signaling mechanisms underlying the activity of ABC transporters in sunitinib-sensitive and sunitinib-resistant human kidney cancer cell lines (CAKI-2 and A-498) would provide a clear understanding of the relationship between ABC transporter activity and the associated intracellular signaling pathways. Additional limitations include the lack of functional transporter inhibition experiments. The present study is primarily correlative in nature. Although altered ABC transporter expression was observed in resistant RCC models, functional validation using transporter inhibitors or gene-silencing approaches was not performed. Therefore, further studies are required to determine the direct contribution of ABC transporters to sunitinib resistance.

Another limitation of the present study is the absence of a non-malignant renal epithelial cell line. Therefore, baseline ABC transporter expression could not be directly compared between normal and malignant renal cells. Future studies including non-malignant renal cell models will help further characterize transporter expression during RCC development. A limitation of this study is the use of a 40 μM sunitinib concentration, which exceeds clinically achievable plasma levels. Therefore, the observed molecular alterations should be interpreted within the context of an experimental in vitro resistance model rather than as a direct simulation of clinical drug exposure.

## Figures and Tables

**Table 1:** Clinicopathology of the studied RCC patients.

Sample Number	Age	Gender	Histological Subtype	Pathological Grade	TNM	Size
1.	48	male	cc. Renocellulare	G2	pT1b	5 cm
2.	45	male	cc. Renocellulare	G2	pT1b	5 cm
3.	59	male	cc. Renocellulare	G2	pT1a	3.5 cm
4.	71	female	cc. Renocellulare	G4	pT2a	9 cm
5.	70	female	cc. Renocellulare	G3	pT1b	6 cm
6.	64	male	cc. Renocellulare	G2	pT1b	5.9 cm
7.	74	female	cc. Renocellulare	G4	pT3a	7.5 cm
8.	80	male	cc. Renocellulare	G4	pT3a	8.7 cm
9.	74	male	Eosinophil cc.	G3	pT1a	3.5 cm
10.	70	male	cc. Renocellulare	G4	T3bm pN0pM1	10 cm
11.	69	male	cc. Renocellulare	G2	pT1a	4 cm
12.	66	male	cc. Renocellulare	G1	pT3a	4.5cm
13.	61	male	cc. Renocellulare	G3	pT1b	4.5 cm
14.	56	male	cc. Renocellulare	G2	pT1b	4.5 cm
15.	67	female	cc. Renocellulare	G2	pT1b	5.6 cm
16.	68	female	cc. Renocellulare	G2	pT1a	2.8 cm
17.	59	female	cc. Renocellulare	G1	pT1b	6.2 cm
18.	52	female	cc. Renocellulare	G3	pT3a pN1	12 cm
19.	67	male	cc. Renocellulare	G3	pT3a	7.7 cm
20.	83	male	cc. Renocellulare	G1	pT1a	2.3 cm

Note. pT1a: tumor size is less than 4 cm and organ localized, pT1b: tumor size is more than 4 cm, but less than 7 cm, and the tumor is organ localized; pT3a: tumor extends into the renal vein or its segmental branches, ccRCC: clear cell renal cell carcinoma; pN0/M1: not spread to regional lymph nodes, but it has spread to distant organs/sites; pN1: micrometastases; or metastases in 1–3 axillary lymph nodes.

## Data Availability

The data presented in this study are available on request from the corresponding author.

## Supplementary Materials

The following supporting information can be downloaded at: https://www.mdpi.com/article/10.3390/cimb48070739/s1.

## Abbreviations

The following abbreviations are used in this manuscript:

RCC	Renal cell carcinoma
mRCC	Metastatic renal cell carcinoma
TKIs	Tyrosine kinase inhibitors
MDR	Multidrug resistance
ABC transporters	ATP binding cassette transporters
ABCB1	ATP binding cassette subfamily B member 1
ABCB5	ATP binding cassette subfamily B member 5
ABCG2	ATP binding cassette subfamily G member 2
ROS	Reactive oxygen species
PI3K	Phosphoinositide 3-kinase
Akt (PKB)	Protein kinase B
MAPK	Mitogen-activated protein kinase
ERK	Extracellular signal-regulated kinase
Wnt	Wingless-Type MMTV Integration Site
ATCC	American Type Culture Collection
IMDM	Iscove’s Modified Dulbecco’s Medium
FBS	Fetal Bovine Serum
DMSO	Dimethyl sulfoxide
cDNA	Complementary DNA
RT-PCR	Reverse transcription polymerase chain reaction
qRT-PCR	Quantitative real-time polymerase chain reaction
GAPDH	Glyceraldehyde 3-phosphate dehydrogenase
SDS-PAGE	Sodium dodecyl sulfate-polyacrylamide gel electrophoresis
PVDF	Polyvinylidene fluoride
TBST	Tris buffered saline with Tween-20
HPRT	Hypoxanthine Phosphoribosyltransferase
mTOR	Mammalian target of rapamycin
pAkt	Phospho-Akt
Bax	Bcl-2-like protein 4
PTEN	Phosphatidylinositol-3,4,5-trisphosphate 3-phosphatase
NF-κB	Nuclear factor kappa-light-chain-enhancer of activated B cells
pERK	Phospho-ERK
