# Supplementary material for: Expression of ABCB1, ABCB5, and ABCG2 Transporters in Human Renal Cell Carcinoma and Their Underlying Signaling Pathways
Source: Curr Issues Mol Biol. 2026 Jul 21;48(7):739. doi: 10.3390/cimb48070739 (PMC13408919; doi:10.3390/cimb48070739)
Supplement: Supplementary file 1 [file cimb-48-00739-s001.zip › cimb-4409569-supplementary.pdf]

Supplementary Figures and Tables to the manuscript

Supplementary Table S1

Table S1: List of the primer sequences used for qRT-PCR.

Primer	Forward	Reverse	Tm
**ABCB1**	5’-GCTGTCAAGGAAGCCAATGCCT-3’	5’-TGCAATGGCGATCCTCTGCTTC-3’	60°C
**ABCB5**	5’-TTTGCCTATGCGGCAGGGTTTC-3’	5’-CAAAACGAGCGTTTCTCCGATGG-3’	60°C
**BCRP1/ ABCG2**	5’-GTTCTCAGCAGCTCTTCGGCTT-3’	5’-TCCTCCAGACACACCACGGATA-3’	60°C
**GAPDH**	5’-TGTAGTTGAGGTCAATGAAGGG-3’	5’-ACATCGCTCAGACACCATG-3’	60°C

Supplementary Table S2

Table S2: List of the antibodies used for Western blot

**Antibody**	**Origin, catalog number**	**Dilution applied**
MDR1/ABCB1 (E1Y7B) Rabbit mAb	Cell Signaling, #13342	1:1000
ABCB5 Polyclonal Antibody	ThermoFisher SCIENTIFIC #PA5-114801	1:1000
ABCG2 (D5V2K) Rabbit mAb	Cell Signaling, #42078	1:1000
PI3 Kinase p110 alpha (C73F8) Rabbit mAb	Cell Signaling, #4249	1:1000
Akt (pan) (C67E7) Rabbit mAb	Cell Signaling, #4691	1:1000
Phospho-Akt (Ser473) (D9E) Rabbit mAb	Cell Signaling, #4060	1:2000
Bax (D2E11) Rabbit mAb	Cell Signaling, #5023	1:1000
PTEN (D4.3) Rabbit mAb	Cell Signaling, #9188	1:1000
NF-kappaB p65 (D14E12) Rabbit mAb	Cell Signaling, #8242	1:1000
p44/42 MAPK (Erk1/2) Rabbit mAb	Cell Signaling, #9102	1:1000
Phospho-p44/42 MAPK (Erk1/2) (Thr202/Tyr204) Rabbit mAb	Cell Signaling, #9101	1:1000
Anti- HPRT1 (P00492) Rabbit Mab	BOSTER Biological Technology #M00668	1:1000

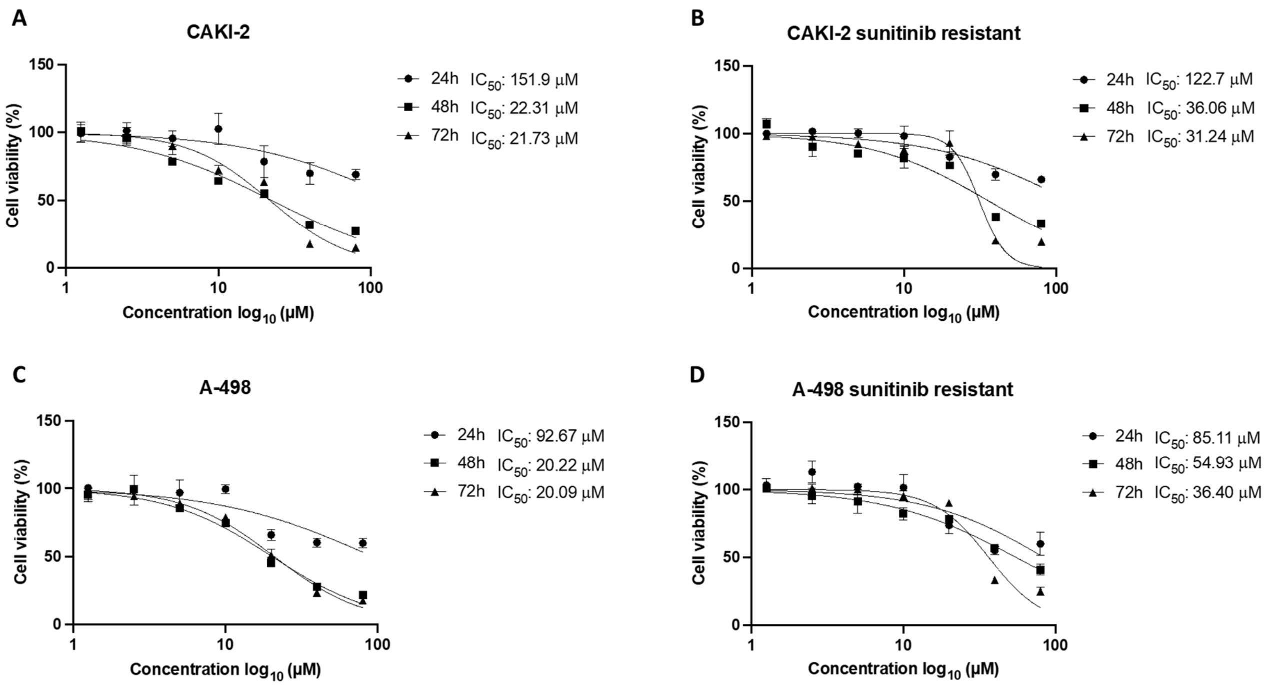

Figure S1: Dose–response analysis of sunitinib in sensitive and resistant renal carcinoma cell lines. CAKI-2, CAKI-2 sunitinib-resistant, A-498 and A-498 sunitinib-resistant cells were treated with increasing concentrations of sunitinib for 24, 48 and 72 h, and cell viability was determined. Dose–response curves were generated and IC_{50} values were calculated for each time point. The calculated IC_{50} values were as follows: CAKI-2 (24 h: 151.9 μM; 48 h: 22.31 μM; 72 h: 21.73 μM), CAKI-2 sunitinib-resistant (24 h: 122.7 μM; 48 h: 36.06 μM; 72 h: 31.24 μM), A-498 (24 h: 92.67 μM; 48 h: 20.22 μM; 72 h: 20.09 μM), and A-498 sunitinib-resistant (24 h: 85.11 μM; 48 h: 54.93 μM; 72 h: 36.40 μM). Based on the 72-hour IC_{50} values obtained for all four cell lines, a treatment concentration of 40 μM sunitinib was selected for subsequent experiments.
